# Efficacy of extracorporeal shockwave therapy in the treatment of postherpetic neuralgia: A pilot study

**DOI:** 10.1097/MD.0000000000019516

**Published:** 2020-03-20

**Authors:** Sung Hyun Lee, Kyoung-Ho Ryu, Pyoung On Kim, Hyo-Won Lee, Eun-Ah Cho, Jin-Hee Ahn, Inyoung Youn, Kyung Seung Yang

**Affiliations:** a Department of Anesthesiology and Pain Medicine; b Department of Radiology, Kangbuk Samsung Hospital, Sungkyunkwan University School of Medicine; c Department of Anesthesiology and Pain Medicine, Seoul St. Mary's clinic, Seoul, Korea.

**Keywords:** extracorporeal shockwave therapy, postherpetic itch, postherpetic neuralgia

## Abstract

Established conventional treatments for postherpetic neuralgia (PHN) and postherpetic itch (PHI) are difficult and often disappointing. In this study, the authors investigated the effect and mechanisms of extracorporeal shockwave therapy (ESWT) on pain and itch associated with PHN and PHI.

Thirteen patients, 50 to 80 years of age, with symptoms associated with PHN or PHI (duration of persistent pain >3 months) and complaints of pain or itch rated >4 on a numerical rating scale (NRS), were included. ESWT was administered using a shockwave device (Piezo Shockwave^2^, Richard Wolf GmbH, Knittlingen, Germany) to skin areas affected by pain or itch. An energy flux density of 0.09 to 0.16 mJ/mm^2^ at a frequency of 5 Hz and 2000 impulses was administered at 3-day intervals for 6 sessions. The NRS, 5D-Itch Scale, and Patients Global Impression of Change (PGIC) scale were used to evaluate the efficacy of ESWT.

NRS scores of pain and itch and 5D-Itch Scale scores decreased significantly compared with before treatment and at the end of the treatment sessions (*P* < .0001, *P* = .001, *P* = .0002, respectively). There was a statistically significant difference between PGIC scores, which were checked every 2 sessions (*P* < .0001).

ESWT is a noninvasive modality that significantly reduced PHN-associated pain and itch.

## 1 Introduction

The most common neurological complications associated with herpes zoster (HZ) is postherpetic neuralgia (PHN), defined as pain lasting for 90 days after the onset of rash. PHN is characterized by neuropathic pain, including a persistent spontaneous sharp or paroxysmal burning sensation, allodynia, and/or hyperesthesia, that affects patient quality of life. Of patients >50 years of age, approximately 10% to 15% with HZ develop PHN, and this complication occurs more often in older patients.^[1–3]^ The pathophysiology of PHN is related to disturbances in the peripheral and central nervous systems. The response to HZ virus-induced nerve inflammation results in anatomical and functional injury to neural tissue in the peripheral nerves, dorsal root ganglion, and spinal cord, resulting in the onset of neuropathic pain and sensory abnormalities in the affected dermatome.^[4–6]^ Patients affected by PHN pain also experience itch, termed postherpetic itch (PHI). The prevalence of PHI is considerable, and affects 9% of patients in the acute stage; moreover, the prevalence of PHI is 30% to 58% at the chronic stage in those who develop PHN.^[7,8]^ PHI is clinically defined as itching concomitant with pain or a sensation of itching that increases as pain subsides. Furthermore, only the itch sensation remains without pain. In such cases, the itching becomes troublesome. PHI also has 2 causes, peripheral and central, similar to PHN.

Extracorporeal shockwave therapy (ESWT) is usually used to treat musculoskeletal diseases such as nonunion fractures, plantar fasciitis, and epicondylitis, among others.^[9,10]^ The principle of shockwave therapy is the production of mechanical energy using pressurized air. This energy is transmitted in the tissues as a longitudinal wave. It contributes to regeneration and revascularization by causing microfunctional and microstructural changes. Low and medium energy levels involve intercell and cell–matrix interactions and modify the cell membrane, and functional changes in cytoplasmic organelles.^[11–13]^ Based on these mechanisms, ESWT can be considered a method of mechanotherapy for analgesia, regeneration, and reduction of neuroinflammation. ESWT can be applied to treat PHN and PHI that are refractory to medication based on their pathophysiology. In other words, ESWT is a safe and noninvasive treatment mainly used in musculoskeletal system, but its application can be extended to PHN expecting to induce pain relief and tissue regeneration.

To our knowledge, no previous studies have applied ESWT to PHN and/or PHI, although several previous investigations have described the efficacy of ESWT in burn pruritus and pain similar to PHI and PHN in terms of sharing a similar neuropathic mechanism.^[14,15]^ The main objective of the present study, therefore, was to evaluate the effect of focused ESWT on patients with PHN or PHI.

## 2 Materials and methods

### 2.1 Participants

This open-label, retrospective pilot study was conducted according to the tenets of the Declaration of Helsinki. The research protocol was approved by the local Institutional Review Board of Kangbuk Samsung Hospital, Seoul, Korea. All subjects were outpatients at a pain clinic of the hospital. Patients 50 to 80 years of age with symptoms appropriate for PHN or PHI (duration of persistent pain >3 months) undergoing classic conservative therapy such as combination medication treatment (pregabalin 150–450 mg, tramadol 75–150 mg, and acetaminophen 650–1300 mg) and nerve block^[16]^ and with complaints of pain or itch with a rating >4 on an numerical rating scale (NRS) were included. Of patients who complained of itch, only those who complained of accompanying pain were included. Patients with contraindications to ESWT, including malignancy or acute infections, or inflammation of the affected area, history of epilepsy, coagulopathies, or cardiac pacemaker, were excluded from this study.

### 2.2 Protocol

Among the affected skin areas with pain or itching, the primary treatment site where the symptoms were reproduced when the probe was placed and impulse and shockwaves were being delivered was selected. ESWT was conducted using a shockwave device (Piezo Shockwave^2^, Richard Wolf GmbH, Knittlingen, Germany) equipped with piezo ceramic crystals for the focused shockwave. Accordingly, treatment was continued by moving the probe to tender areas in a sequential manner at a distance of 40 to 50 mm, which was set considering focal zone (46 mm × 4 mm) of linear, focused, piezo source after ESWT at 100 impulses/cm^2^ on the primary treatment site. The energy flux density was 0.09 to 0.16 mJ/mm^2^; with a frequency of 5 Hz and 2000 impulses were administered at 3-day intervals for 6 sessions.

### 2.3 Outcome assessment

To evaluate the effect of ESWT on PHN, a NRS was used to assess the degree of pain at every treatment session. Additional efficacy measures included the Patients Global Impression of Change (PGIC), a patient rated instrument measuring change in overall patient status on a scale from 1 (very much improved) to 7 (very much worse) at weekly intervals, every 2 sessions.^[17]^

To evaluate the effect of ESWT on PHI, the NRS was used to evaluate the degree of subjective pruritus before treatment and immediately after every 2 sessions: 0 represented no pruritus and 10 represented the worst possible itching. The 5-D Itch Scale, consisting of 5 dimensions, was used. Each item was rated from 1 to 5 points according to the degree of pruritus, and the total score was calculated to quantify pruritus. 5-D scores can potentially range from 5 (no pruritus) to 25 (most severe pruritus).^[15,18]^

### 2.4 Statistical analysis

Data are expressed as mean ± standard error of the mean. Statistical analyses were performed using GraphPad Prism version 5.0 (GraphPad Software, Inc, San Diego, CA). Repeated measures analysis of variance was used to compare NRS scores of pain, pruritus before treatment, and after every session (first session to the sixth session), followed by Bonferroni post hoc tests. PGIC and 5-D scores also were analyzed using repeated measures analysis of variance with the factor time (after 1–3 weeks). Comparisons between NRS before treatment and NRS at every session value were performed at each time point using the Student *t* test and Bonferroni post hoc tests. All statistical tests were 2-tailed and *P* < .05 was considered to be statistically significant.

## 3 Results

**Figure 1:**
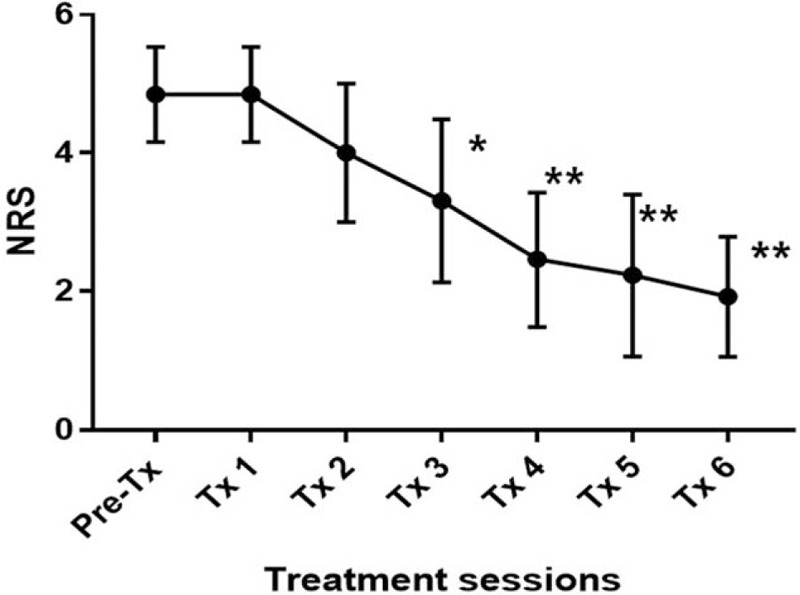
Changes in NRS of pain after treatment. ^∗^, *P* < .005; ^∗∗^, *P* < .0001 when compared with NRS before treatment. NRS = numerical rating scale, Pre-Tx = pretreatment.

**Figure 2:**
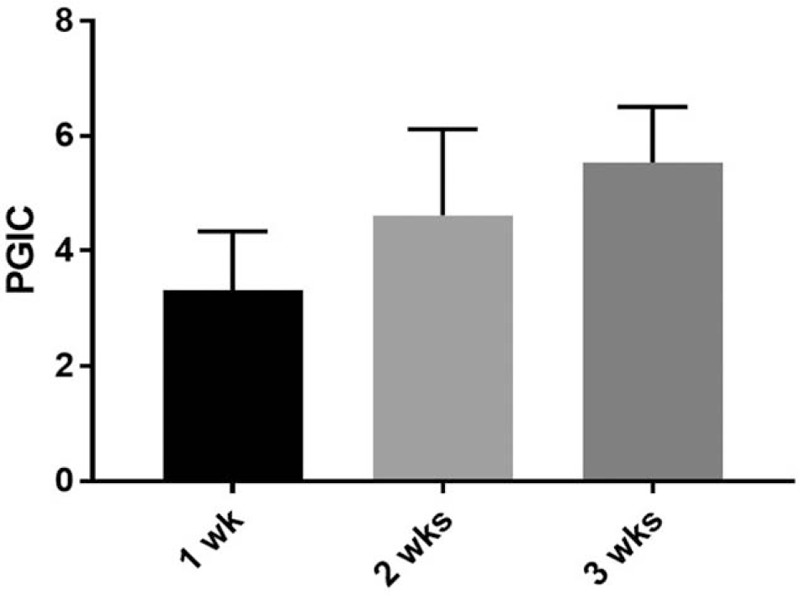
Changes of PGIC score after treatment. ^∗^, *P* < .0005; ^∗∗^, *P* < .0001 when compared with PGIC score before treatment. NRS = numerical rating scale, PGIC = Patients Global Impression of Change.

**Figure 3:**
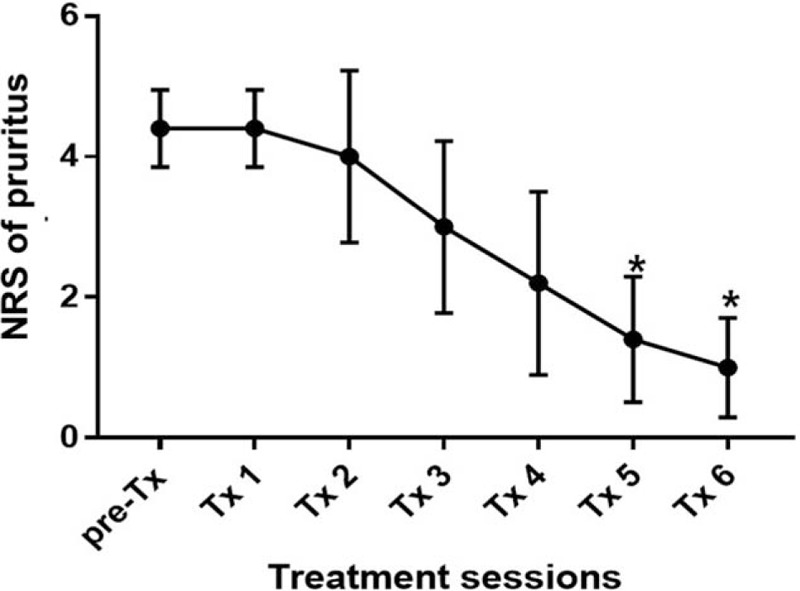
Changes in NRS of pruritus after treatment. ^∗^, *P* < .01 when compared with NRS before treatment. NRS = numerical rating scale, Pre-Tx = pretreatment.

**Figure 4:**
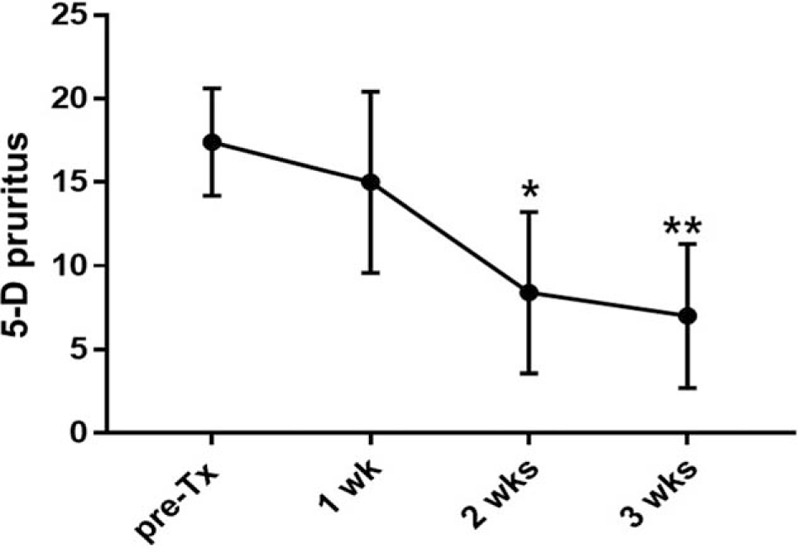
Changes in 5-D pruritus score after treatment. ^∗^, *P* < .05; ^∗∗^, *P* < .01 when compared with 5-D pruritus score before treatment. Pre-Tx = pretreatment.

Thirteen patients participated in the present study. Patient demographic information (sex, age, duration of symptoms, affected dermatome) is summarized in Table 1. There was a statistically significant difference in NRS score at each session (F [3.334, 40] = 41.32; *P* < .0001) (Fig. 1). The time when the comparison between the NRS before treatment and the NRS score for each session after treatment to show a statistically significant difference was after 3 sessions (*P* = .008). There was statistically significant difference in NRS scores at the 2 points when comparing the third session of ESWT and sixth session (*P* = .010). There was a statistically significant difference between PGIC scores, which were checked every 2 sessions (F [3, 44] = 11.29; *P* < .0001) (Fig. 2). Differences in NRS scores for pruritus were statistically significant after each session (F [1.729, 6.914] = 20.63; *P* = .001) (Fig. 3). The time when the comparison between the NRS score for pruritus before treatment and the NRS for pruritus for each session after treatment to demonstrate a statistically significant difference was 5 sessions (*P* = .007). Differences in 5-D Itch Scale scores were statistically significant (F [1.93, 7.719] = 31.58; *P* = .0002) (Fig. 4). The 5-D Itch Scale scores were significantly decreased after 4 sessions (*P* = .013).

**Table 1:**
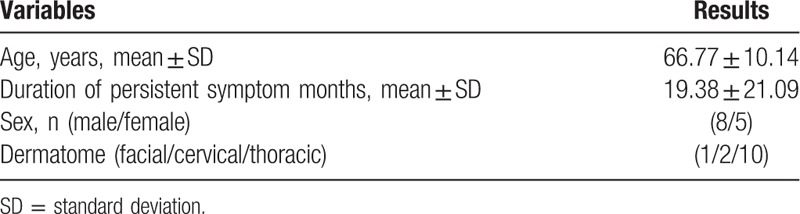
Demographic data of participants.

## 4 Discussion

This study demonstrated that ESWT significantly reduced pain and severity of pruritus in patients with PHN. Patients with PHN complain of decreased quality of life and disturbances with the activities of daily living. PHN is difficult to treat due to its complex pathophysiology, which remains to be clarified. The concomitant inflammatory reaction of the dorsal root ganglion, peripheral nerve, and nerve endings on skin damage presumably lead to HZ-associated pain. This tissue damage and cascading inflammation stimulates nociceptors, causing excitatory and repetitive painful stimuli resulting in central sensitization of the nociceptive system. Initially, replicating HZ particle in infected neurons can be result to direct neurolytic lesions to the cell bodies or their axons, and indirect injury by the endoneurial inflammatory response and hemorrhage. Damage in axonal membrane and cell body can generate continue episodes of injury discharge. Injury discharge from afferent C fiber endings in the affected skin will release the various neurotransmitters onto spinal cord dorsal horn neurons which initiate and develop central sensitization in associated spinal segments. These processes are considered to be the most important mechanism underlying the chronic pain associated with PHN.^[5,19–21]^ Resembling PHN, PHI is associated with neuronal damage. One study reported profound damage, describing a loss of up to 96% of epidermal innervations, in other words, peripheral sensory neurons in the itching area.^[22]^ This may result in hyperactivity of hypoafferented central itch-specific neurons, selective preservation of peripheral itch fibers from unaffected adjacent dermatomes, or excitation of second-order sensory neurons. However, the exact mechanism of PHI is not well understood; peripheral sensitization (increased reaction of primary sensory neurons to itch mediators), central sensitization (hyperactivity of spinal connecting neurons and excitatory interneurons), loss of descending control in the spinal cord, and neuroimmune and neuroglial interactions have been suggested as possibilities.^[7,23,24]^ Considering these mechanisms, treatment of PHN and PHI is required to suppress various neurotransmitters associated with central sensitization to promote regeneration of damaged tissues.

Shockwaves were originally introduced in medicine for kidney stone treatment as extracorporeal shockwave lithotripsy, and clinical applications have since spread. ESWT has significantly expanded, first to musculoskeletal diseases and, later, to regenerative medicine. Shockwaves are mechanical in nature, and consist of an initial positive very rapid phase, exhibiting high amplitude, followed a few microseconds later, by a sudden phase of mild negative pressure, afterward returning to basic values. Shockwaves are mechanical waves, which act as the physical stimulus to correlate the interactions of physical energies with various tissues and cellular elements. In previous studies, ESWT as mechanotherapy, was demonstrated to positively influence some cellular functions and local homeostasis, therefore leading to tissue building and self-healing capabilities.^[25,26]^ The effects of shockwaves on wound healing, bone regeneration, and skin grafts have also been extensively studied. However, no studies have yet applied ESWT to neuropathic symptoms in PHN or PHI. Several studies have investigated the effect of ESWT on burn scar itch and pain with aspects of neuropathic symptoms. These studies have noted that ESWT can cause alterations in the target cells. These interactions involve intracell and cell–matrix interactions. The production of proteins, nitric oxide, and specific growth factors contribute to the activation of the biological process, which results in achieving tissue regeneration. ESWT applied to peripheral nerve injury induced analgesia accompanied by removal of the degenerated axons and increases the capacity of the injured axons to regenerate. ESWT revealed an important role in the neural regeneration through increasing the myelin sheath proliferation, axons regeneration consequently significant enhancement of the nerve functional recovery.^[27–29]^ In a similar context, ESWT can be considered for the treatment of PHN and PHI.

## 5 Limitations

The present investigation included only a limited number of subjects in the preliminary study and no control group. In addition, there was no precedent study and, as such, there is a lack of evidence regarding how many sessions are needed. Nevertheless, the present study demonstrated improvement in symptoms after ≥3 sessions, and revealed that the effect increased as the number of treatment sessions increased to 6. However, because it is targeted at a small number, comparative studies are also needed to determine the optimal number of treatments for larger subjects. There is also a limit to how long the therapeutic effects of ESWT will remain in this study because of short-term follow-up.

## 6 Conclusion

To our knowledge, this is the first report to describe the use of ESWT in PHN patients with or without PHI. We found that ESWT was clinically useful as a noninvasive therapy for pain and itch associated with PHN, and can be considered an effective alternative modality for the treatment of PHN and PHI.

## Author contributions

**Conceptualization:** Sung Hyun Lee, Eun-Ah Cho, Kyung Seung Yang.

**Data curation:** Sung Hyun Lee, Hyo-Won Lee, Pyoung On Kim.

**Writing–original draft:** Sung Hyun Lee, Jin-Hee Ahn.

**Writing–review & editing:** Sung Hyun Lee, Kyoung-Ho Ryu.

**Supervision:** Kyoung-Ho Ryu.

**Formal analysis:** Eun-Ah Cho.

**Validation:** Inyoung Youn.

**Investigation:** Jin-Hee Ahn.

Sung Hyun Lee orcid: 0000-0001-5616-6649.
